# Digital Health Technology Use Among Spanish Speakers in the US: A Scoping Review

**DOI:** 10.1001/jamanetworkopen.2025.10386

**Published:** 2025-05-15

**Authors:** Robin T. Higashi, Bhaskar Thakur, Emily C. Repasky, Alejandra Casillas, Bryan D. Steitz, Timothy P. Hogan, Christoph U. Lehmann, Eric D. Peterson, Ann Marie Navar, Robert W. Turer

**Affiliations:** 1 O’Donnell School of Public Health, The University of Texas Southwestern Medical Center, Dallas; 2 Department of Family and Community Medicine, The University of Texas Southwestern Medical Center, Dallas; 3 Department of Emergency Medicine, The University of Texas Southwestern Medical Center, Dallas; 4 Department of Physical Medicine and Rehabilitation, Peter O’Donnell Jr. Brain Institute, The University of Texas Southwestern Medical Center, Dallas; 5 Division of General International Medicine and Health Services Research, David Geffen School of Medicine, University of California, Los Angeles; 6 Department of Biomedical Informatics, Vanderbilt University Medical Center, Nashville, Tennessee; 7 eHealth Partnered Evaluation Initiative, VA Bedford Healthcare System, Bedford, Massachusetts; 8 Center for Health Optimization and Implementation Research, VA Bedford Healthcare System, Bedford, Massachusetts; 9 Clinical Informatics Center, The University of Texas Southwestern Medical Center, Dallas; 10 Department of Internal Medicine, The University of Texas Southwestern Medical Center, Dallas; 11 Deputy Editor, *JAMA Cardiology*

## Abstract

**Importance:**

Digital health technologies include patient portals, telehealth, mobile health, and web-based resources; they have the potential to expand health care access, increase quality of care, and improve health outcomes. An emerging literature describes factors associated with disparities between Spanish and English speakers with the use of digital health tools and documents.

**Objective:**

To characterize barriers and facilitators and to inform hypothesis-generating questions and intervention planning associated with digital health technology use among Spanish-speaking populations in the US.

**Evidence Review:**

Between January 2023 and April 2024, PubMed, Scopus, Web of Science, and Google Scholar were searched for publications between January 2013 and April 2024. Systematic reviews, protocols, editorials, abstracts, unpublished literature, non–peer-reviewed literature, and non–US-based and non-English studies were excluded. The Covidence platform was used to avoid duplicate records, and an abstract and full-text screening were then conducted for exclusions. Unstructured text in the final dataset was thematically analyzed.

**Findings:**

Of 688 publications searched, 192 were excluded as duplications, 277 were excluded from abstract screenings, and 113 were excluded from full-text screenings. Among the 106 included studies (68% of which were published between 2019 and 2024), 73 (69%) used quantitative methods, 15 (14%) used qualitative methods, and 18 (17%) used applied mixed methods. The primary technologies studied were characterized as portal (21% [n = 22]), telehealth (42% [n = 45]), mobile health (16% [n = 17]), web-based resources (9% [n = 10]), and mixed (studies reporting >1 technology; 11% [n = 12]). Compared with English speakers, studies revealed consistently lower portal account activation, portal use, telehealth adoption, and online resource use among Spanish speakers. Barriers to use across all technologies included limited access to technology and Wi-Fi, low literacy, and limited digital literacy. Barriers to portal and telehealth use included lack of awareness of digital tools, limited patient-facing instructions in Spanish, and interpreter-related challenges. Facilitators cited across multiple technologies included use of text messaging and social media interventions, interventions involving care partners and/or interpreters, and culturally and linguistically tailored Spanish materials.

**Conclusions and Relevance:**

The results of this scoping review suggest that Spanish-speaking persons in the US faced technology, language, and literacy-related barriers to digital technology use. Embracing support for care partners, facilitating text message or social media–oriented workflows, and ensuring that materials are linguistically and culturally tailored represent approaches for health systems, electronic health record vendors, and community health organizations to mitigate these disparities.

## Key Points

**Question:**

What factors are associated with digital health technology use among Spanish speakers in the US?

**Findings:**

In this scoping review of 106 studies, 68% of which were published between 2019 and 2024, disparities in digital health technology use between Spanish and English speakers were summarized. Barriers for Spanish speakers included limited consistent access to wireless networking technology, lack of overall and digital literacy, and lack of awareness, while facilitators included integrating interpreters, care partners, culturally tailored materials, and text messaging–based or application-based interventions.

**Meaning:**

The findings of this study suggest that barriers to digital health technology may exacerbate existing health outcome disparities; however, interventions that incorporate facilitators may promote digital health equity.

## Teaser

This scoping review summarizes factors that are associated with digital health technology use among Spanish speakers in the US.

## Introduction

Digital health technologies include patient portals, telehealth, web-based resources, and mobile health (mHealth) applications. These technologies have been associated with expanded health care access, increased care quality, reduced acute care visits, and improved health outcomes among mostly English-speaking populations.^1,2,3,4,5,6,7,8,9,10,11,12,13,14,15,16,17,18,19,20^ However, the benefits have been unrealized among some medically underserved communities, notably patients with limited English proficiency (LEP), who have less access to care and worse health outcomes.^21,22^ The 40 million individuals who primarily prefer to speak Spanish (hereinafter Spanish speakers) comprise the largest US LEP population, and they face substantial barriers to care that may differ from other LEP populations.^23,24^

Studies from the COVID-19 pandemic highlight disparities associated with awareness, enrollment, and use of digital health technologies among Spanish speakers.^25,26,27^ However, widespread awareness of the problem and effective strategies to address identified barriers are limited. Spanish speakers face multiple digital health barriers, partially associated with low digital literacy, linguistic barriers, and limited broadband access.^24,27,28,29^ Some Spanish speakers may face heightened cultural and socioeconomic barriers that create hesitancy to disclose personal information through digital devices. Inequitable digital health access can exacerbate health disparities, which suggests that digital health engagement is a priority for this population.^30,31^

Scoping reviews map the breadth of literature on a particular topic to identify core concepts, strategies, and outcomes from existing literature to inform future research, interventions, and guidelines.^32^ The objective of this scoping review is to identify and map the existing literature regarding the patterns, barriers, and facilitators of digital health technology use among Spanish speakers in the US.

## Methods

This scoping review protocol followed the Preferred Reporting Items for Systematic Reviews and Meta-Analyses Extension for Scoping Reviews (PRISMA-ScR) reporting guideline and checklist (eTable 1 in Supplement 1). The review did not require institutional review board approval because it was not human participant research and used only publicly available data.

### Literature Search

Between January 2023 and April 2024, we queried PubMed, Scopus, Wed of Science, and Google Scholar, guided by our research question: What are the current use patterns, barriers, and facilitators associated with digital health technology use among Spanish speakers in the US? We searched Google Scholar using the terms *Spanish* and *patient portal*, which identified 2 unrelated reviews, confirming the novelty of the topic. We then searched PubMed using the terms *Spanish* and *patient portal* and reviewed the first 41 resulting articles to identify key search terms and MeSH (Medical Subject Headings) terms. We expanded our search to add other digital health technologies because the literature on portals was limited and often addressed multiple technologies. Final PubMed search terms, which we converted for use in Scopus and Web of Science, are presented in eTable 2 in Supplement 1. We also included the first 50 results from our original exploratory search in Google Scholar. The search included literature from January 2013 through April 2024.

### Study Screening and Data Extraction

We managed data using Covidence (Veritas Health Innovation). During the abstract and full-text screening, studies were reviewed independently by 2 team members; discrepancies were resolved by consensus using a third study member. Analyses were performed by 4 team members (R.T.H., B.T., E.C.R., and R.W.T.), 2 of whom were assigned to each study in a matrix pattern to reduce bias. We included US-based peer-reviewed studies, excluding nonoriginal research studies. Complete inclusion and exclusion criteria and definitions of terms are included in the PRISMA diagram (Figure) and the Covidence extraction template (eFigure in Supplement 1). For 6 studies that met initial screening criteria, but Spanish outcome measures were not described, we emailed corresponding authors to request clarification. Five authors responded with necessary clarifications, and all 5 studies were included; the sixth study was excluded. Two reviewers independently extracted data using the extraction form, which was informed by existing health equity scoping reviews (eFigure in Supplement 1).^33,34^ We consolidated each pair of extracted data using consensus review by 3 group members.

**Figure.**
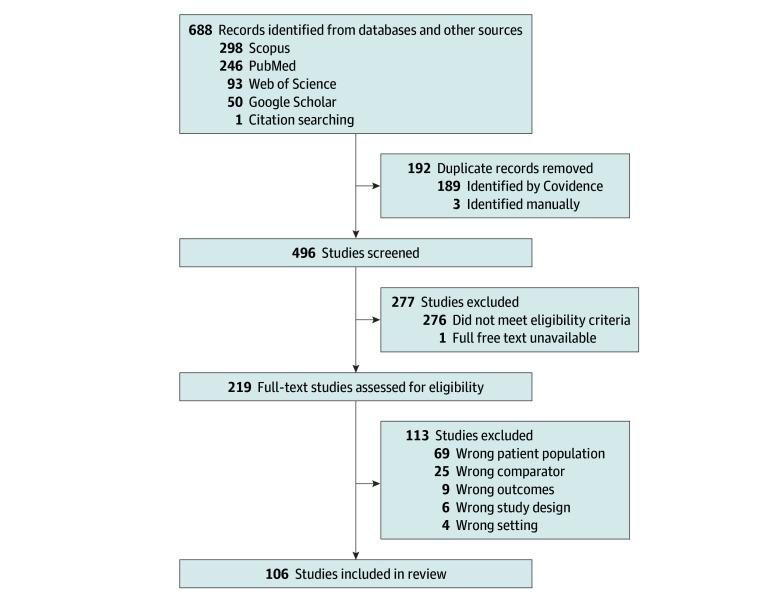
Flowchart of Included Studies Inclusion criteria included peer-reviewed literature relevant to the research question. Exclusion criteria included systematic reviews, protocols, editorials, case studies, viewpoints, abstracts, and unpublished literature (wrong study design); studies with non-US populations or not written in English (wrong setting); studies in which Spanish-speaking patients were not included, or the Spanish-speaking population was not quantified (wrong patient population); studies in which the Spanish-speaking population was quantified, but either an analysis by language was not performed, or an analysis by language was performed, but no findings or inferences were stated (eg, the Spanish-speaking population may have been quantified, but the outcome was analyzed by English vs non-English languages in aggregate rather than by a unique language) (wrong comparator); and studies in which digital health technology was not the focus (eg, an intervention intended to occur by telehealth occurred mostly asynchronously by telephone [wrong outcomes]). If the full free text was not available, after contacting the corresponding authors, the studies were also excluded.

### Thematic Analysis and Descriptive Statistics

Reviewers trained in qualitative analyses (R.T.H. and E.C.R.) supervised thematic coding of unstructured extracted data using rapid analysis, resolving discrepancies through discussion. We created qualitative codebooks (eTables 3 and 4 in Supplement 1) to analyze unstructured databases on a review of a randomized 20% sample of the data for each variable. The final codebook was applied to all extracted studies to identify use patterns, outcomes, barriers, and facilitators described in study objectives and key findings. Unstructured descriptions of study populations were thematically analyzed using the following variables, if specified: sociodemographic characteristics, setting, illness, and technology exposure.

We coded all structured data according to definitions in the eFigure in Supplement 1. We also assigned a primary technology (patient portal, telehealth, mHealth, web resources, or mixed [studies reporting >1 technology]) to facilitate descriptive statistics. After thematic coding and categorization, we counted the studies by design, care setting, study population, and region, stratified by technology. We computed the medians and IQRs of total and Spanish-speaking sample sizes, stratified by study design (Table 1). Analyses were conducted using R, version 4.4.2 (R Project for Statistical Computing).

**Table 1.** Sample Size by Study Methods Used

Sample	Study design, median (IQR)		
	Qualitative (n = 15)	Quantitative (n = 73)	Mixed methods (n = 18)
Total, No.	27 (20-34)	2020 (241-17 400)	54 (40-409)
Proportion of Spanish speakers, %	89 (41-100)	19 (8-49)	64 (28-92)

## Results

Our initial search yielded 688 studies. After removing 192 duplicates, we screened 496 abstracts. Abstract screening (n = 277) yielded 219 studies eligible for full-text review. After full-text review (n = 113), 106 studies, among which 72 (68%) were published within the past 5 years, met inclusion criteria and underwent full extraction and analysis (Figure).

Table 2 describes the 106 included studies, stratified by technology. The primary technologies studied were characterized as portal (21% [n = 22]), telehealth (42% [n = 45]), mHealth (16% [n = 17]), web-based resources (9% [n = 10]), and mixed (11% [n = 12]). In most studies, 76 (72%) focused on adult care settings and 96 (91%) on patients or care partners; 73 (69%) used quantitative methods, and 18 (17%) applied mixed methods. Most portal studies (20 of 22 [91%]) and telehealth studies (34 of 45 [76%]) used quantitative methods, while 10 of 17 mHealth studies (59%) and 6 of 10 web-based resource studies (60%) used primarily qualitative or mixed methods designs. Table 1 reports the median (IQR) overall sample size, stratified by study methods used. Table 3 reports study-level characteristics including study design, geographic region, sample size (overall and Spanish speakers), and technology.

**Table 2.** Study Characteristics Stratified by Technology^a^

Characteristic	Technology					
	Overall (N = 106)	mHealth (n = 17)^b^	Portal (n = 22)^c^	Telehealth (n = 45)^d^	Web-based resources (n = 10)^e^	Mixed (n = 12)^f^
Study design						
Qualitative	15 (14)	5 (29)	0	6 (13)	2 (20)	2 (17)
Quantitative	73 (69)	7 (41)	20 (91)	34 (76)	4 (40)	8 (67)
Mixed methods	18 (17)	5 (29)	2 (9)	5 (11)	4 (40)	2 (17)
Care setting						
Adult	76 (72)	15 (94)	19 (86)	24 (53)	9 (90)	9 (75)
Pediatric	23 (22)	1 (6)	3 (14)	15 (33)	1 (10)	3 (25)
Mixed	6 (6)	0	0	6 (13)	0	0
Unknown	1 (1)	1 (1)	0	0	0	0
Population						
Health professional	2 (2)	1 (6)	0	1 (2)	0	0
Patient	96 (91)	14 (82)	21 (95)	40 (89)	9 (90)	12 (100)
Mixed	8 (8)	2 (12)	1 (5)	4 (9)	1 (10)	0
Region						
Midwest	11 (11)	4 (24)	0	7 (16)	0	0
Northeast	23 (23)	3 (18)	2 (10)	12 (27)	4 (44)	2 (17)
South	25 (25)	4 (24)	8 (40)	7 (16)	2 (22)	4 (33)
West	38 (37)	6 (35)	10 (50)	15 (34)	2 (22)	5 (42)
Multiple regions	7 (7)	0	0	3 (7)	1 (11)	1 (8)
Unknown	2 (2)	0	2 (9)	1 (2)	1 (10)	0
Proportion of total technologies, %	100	16	21	42	9	11

Abbreviation: mHealth, mobile health.

^a^ Data are presented as No. (%) of technology used unless otherwise indicated.

^b^ Included mobile applications (apps), text messaging, chat, digitally connected home health monitoring devices, social media, and telemonitoring.

^c^ When specified, included 21 instances of Epic; 2, Cerner; 1, Athena; and 1, custom (Kaiser).

^d^ Included synchronous video visits.

^e^ Included health-related websites, e-newsletters, and online learning modules.

^f^ Included more than 1 technology (eg, portal and telehealth).

**Table 3.** Characteristics of Included Studies

Source	Research design	US region	Study start date	Study end date	Sample size, No.		Technology studied				
					Overall	Spanish speakers^a^	mHealth^b^	Portal^c^	Tele-health^d^	Web-based^e^	Mixed^f^
Adeli and Bloom,^36^ 2021	Quantitative	Midwest	3/1/2020	6/1/2020	17	8	No	No	Yes	No	No
Allison et al,^37^ 2023	Quantitative	South	10/1/2020	2/28/2021	122	15	No	No	Yes	No	No
Amin et al,^38^ 2020	Quantitative	West	8/1/2018	9/30/2018	566	61	No	No	No	No	Yes
Aponte and Nokes,^39^ 2017	Mixed methods	Northeast	NA	NA	20	16	No	No	No	Yes	No
Arana-Chicas et al,^40^ 2022	Quantitative	Northeast, Midwest	8/1/2018	3/31/2021	457	324	No	No	No	No	Yes
Arora et al,^28^ 2016	Quantitative	West	1/1/2011	12/31/2014	2144	1534	No	No	No	No	Yes
Banas et al,^41^ 2017	Qualitative	Midwest	NA	NA	62	59	Yes	No	No	No	No
Bender et al,^42^ 2016	Qualitative	West	11/1/2012	2/28/2013	21	21	Yes	No	No	No	No
Berry et al,^43^ 2015	Qualitative	Northeast	4/1/2010	7/1/2011	7	7	No	No	No	Yes	No
Blundell et al,^44^ 2021	Quantitative	Northeast	3/18/2020	7/31/2020	1078	53	No	No	Yes	No	No
Burner et al,^45^ 2018	Mixed methods	West	NA	NA	44	26	Yes	No	No	No	No
Bush et al,^46^ 2018	Quantitative	West	1/1/2010	5/31/2016	10 464	1704	No	Yes	No	No	No
Caballero et al,^47^ 2022	Mixed methods	South	NA	NA	30	25	Yes	No	No	No	No
Casillas et al,^48^ 2019	Mixed methods	West	6/1/2017	7/31/2017	46	23	No	Yes	No	No	No
Casillas et al,^49^ 2021	Quantitative	West	3/1/2015	3/31/2019	55 190	9187	No	Yes	No	No	No
Chen et al,^50^ 2021	Quantitative	West	5/2/2019	12/31/2019	273	90	No	No	No	No	Yes
Chuang et al,^51^ 2024	Qualitative	West	9/1/2020	2/28/2021	45	14	No	No	Yes	No	No
Cockrell et al,^52^ 2023	Quantitative	West	4/1/2019	6/30/2020	3334	258	No	No	Yes	No	No
Culhane-Pera et al,^53^ 2023	Mixed methods	Midwest	3/1/2018	4/30/2018	50	26	Yes	No	No	No	No
Dahne et al,^54^ 2019	Quantitative	South	9/1/2017	6/30/2018	42	42	Yes	No	No	No	No
DeCamp et al,^55^ 2023	Mixed methods	West	4/1/2020	7/31/2021	102 328^g^ and 25 qualitative	4298	No	No	Yes	No	No
Cerda Diez et al,^56^ 2019	Mixed methods	Northeast	NA	NA	56	56	No	No	No	Yes	No
Fatehi et al,^57^ 2020	Quantitative	South	10/1/2017	12/31/2019	4676	1015	No	Yes	No	No	No
Fennell et al,^58^ 2022	Quantitative	South	4/1/2013	2/29/2016	39 866	8602	No	No	Yes	No	No
Fernandez et al,^59^ 2017	Quantitative	Northeast	12/1/2012	3/31/2013	200	98	No	Yes	No	No	No
Fischer et al,^60^ 2019	Quantitative	West	10/1/2015	4/30/2018	1518	512	Yes	No	No	No	No
Flower et al,^61^ 2021	Qualitative	South	9/1/2016	12/31/2016	28	28	No	No	No	No	Yes
Fortmann et al,^62^ 2017	Quantitative	West	10/1/2012	2/28/2014	126	116	Yes	No	No	No	No
Frydman et al,^63^ 2022	Quantitative	Northeast	3/1/2020	12/30/2020	491	41	No	No	Yes	No	No
Futterman et al,^64^ 2021	Quantitative	Northeast	3/1/2020	5/1/2020	104	56	No	No	Yes	No	No
Garrido et al,^65^ 2015	Quantitative	Nationwide	2006	12/31/2010	731 108	209 873	No	Yes	No	No	No
Ghaddar et al,^66^ 2020	Quantitative	South	7/23/2018	7/27/2018	322	196	No	No	Yes	No	No
Glassman et al,^67^ 2023	Quantitative	Northeast	4/1/2020	12/31/2021	2023	1006	No	No	Yes	No	No
Guo et al,^68^ 2018	Mixed methods	West	5/1/2015	7/31/2015	26	18	No	Yes	No	No	No
Guzman et al,^69^ 2022	Mixed methods	Midwest	1/1/2018	1/31/2018	52	7	Yes	No	No	No	No
Hamilton et al,^70^ 2018	Quantitative	South	6/1/2016	7/31/2016	171	42	Yes	No	No	No	No
Hsiao et al,^71^ 2021	Quantitative	Midwest	6/1/2020	8/31/2020	197 076	2559	No	No	Yes	No	No
Hsueh et al,^72^ 2023	Quantitative	West	3/16/2020	10/31/2020	13 764	9507	No	No	Yes	No	No
Hundal et al,^73^ 2022	Quantitative	Northeast	5/1/2020	11/30/2020	241	79	No	No	Yes	No	No
Jacobs et al,^74^ 2014	Mixed methods	South	NA	NA	25	25	No	No	Yes	Yes	No
Jelinek et al,^75^ 2022	Quantitative	Midwest	4/1/2020	11/30/2020	98 357	14 596	No	No	Yes	No	No
Jiang et al,^76^ 2021	Quantitative	West	3/23/2019	5/1/2020	3478	529	No	No	Yes	No	No
Kafashzadeh et al,^77^ 2023	Quantitative	West	3/19/2019	3/19/2022	11 656	390	No	Yes	No	No	No
Kim et al,^78^ 2023	Quantitative	West	4/1/2021	6/30/2022	50	34	Yes	No	No	No	No
Kim et al,^79^ 2024	Quantitative	West	1/1/2019	9/30/2020	220	128	No	No	Yes	No	No
Kim et al,^80^ 2024	Quantitative	West	6/1/2020	3/31/2022	19 503	894	No	No	Yes	No	No
Kronforst et al,^81^ 2023	Quantitative	Midwest	3/21/2020	3/17/2021	13 655	1290	No	No	Yes	No	No
Kurth et al,^82^ 2016	Mixed methods	Northeast	Last week of March 2010	2/28/2012	494 RCTs and 77 qualitative	494	No	No	No	Yes	No
Lee et al,^83^ 2022	Qualitative	West	NA	NA	54	54	No	No	No	No	Yes
Localio et al,^84^ 2022	Quantitative	Northeast	7/17/2014	6/30/2017	301	52	No	Yes	No	No	No
Lyles et al,^85^ 2013	Quantitative	West	NA	NA	278	53	Yes	No	No	No	No
Manganello et al,^86^ 2016	Quantitative	Northeast	8/8/2013	11/4/2013	1350	79	No	No	No	No	Yes
Marcus et al,^87^ 2016	Quantitative	West	1/1/2011	12/31/2014	205	199	No	No	No	Yes	No
Marshall et al,^88^ 2023	Qualitative	Northeast	6/1/2021	11/30/2021	10	2	No	No	Yes	No	No
Martinez et al,^89^ 2023	Qualitative	West	Fall 2020	Spring 2021	20	12	No	No	Yes	No	No
Millar et al,^90^ 2020	Quantitative	Nationwide	Public use files of the 2012/2014 program	NA	700	371	No	No	No	Yes	No
Miller et al,^91^ 2024	Quantitative	South	2/1/2017	4/30/2021	443	157	No	No	No	No	Yes
Mook et al,^92^ 2018	Quantitative	South	7/1/2014	6/30/2015	387 198	24 872	No	Yes	No	No	No
Mougey et al,^93^ 2023	Quantitative	Northeast, South	1/1/2019	12/31/2020	17 429	821	No	No	Yes	No	No
Muroff et al,^94^ 2017	Quantitative	Northeast	12/1/2013	7/31/2016	79	79	Yes	No	No	No	No
Natori et al,^95^ 2023	Quantitative	South	10/1/2019	1/31/2022	9553	3486	No	Yes	No	No	No
Nguyen-Grozavu et al^96^ 2023	Qualitative	West	3/1/2021	6/30/2021	27	24	No	No	Yes	No	No
Ochoa et al,^97^ 2017	Quantitative	West	6/1/2014	6/30/2014	459	213	No	Yes	No	No	No
Osei et al,^98^ 2023	Quantitative	Northeast	1/1/2019	12/31/2021	10 848	443	No	No	Yes	No	No
Owolo et al,^99^ 2023	Quantitative	South	1/1/2019	6/30/2021	7955	45	No	Yes	No	No	No
Pack et al,^26^ 2024	Mixed methods	Midwest	6/1/2022	10/31/2022	500	246	No	No	Yes	No	No
Parra-Cardona and DeAndrea,^100^ 2016	Quantitative	Nationwide	1/1/2004	12/31/2010	39 630	12 266	No	No	No	Yes	No
Payvandi et al,^101^ 2022	Mixed methods	Northeast	10/1/2020	3/31/2021	142	30	No	No	Yes	No	No
Pekmezaris et al,^102^ 2016	Qualitative	Northeast	NA	NA	17	1	Yes	No	No	No	No
Pekmezaris et al,^103^ 2020	Qualitative	Northeast	1/1/2019	9/30/2019	35	12	Yes	No	No	No	No
Pelayo et al,^104^ 2023	Qualitative	Midwest	7/1/2022	11/30/2022	20	17	No	No	Yes	No	No
Petros De Guex et al,^105^ 2023	Qualitative	South	2/1/2021	3/31/2021	20	20	Yes	No	No	No	No
Plombon et al,^106^ 2023	Mixed methods	Northeast	7/1/2020	3/31/2022	445	20	No	No	No	No	Yes
Puthenpura et al,^107^ 2021	Quantitative	Northeast	3/17/2020	5/31/2020	1273	69	No	No	Yes	No	No
Qian et al,^108^ 2022	Quantitative	West	1/1/2020	9/30/2020	29 421	2489	No	No	Yes	No	No
Reinosa Segovia and Benuto,^109^ 2024	Quantitative	West	1/1/2021	2/28/2022	25	25	No	No	Yes	No	No
Reuland et al,^110^ 2021	Quantitative	South	2/1/2016	10/31/2016	157	157	No	No	No	No	Yes
Rollison et al,^111^ 2023	Quantitative	South	1/1/2009	12/31/2020	109 816	3162	No	Yes	No	No	No
Rosenthal et al,^112^ 2021	Mixed methods	West	3/11/2020	11/30/2020	5464^g^ and 16 qualitative	407	No	No	Yes	No	No
Rowland et al,^113^ 2022	Mixed methods	Midwest	5/1/2019	12/31/2019	70	60	Yes	No	No	No	No
Roy et al,^114^ 2021	Quantitative	West	1/1/2013	12/31/2018	1849	69	No	Yes	No	No	No
Sadauskas et al,^115^ 2024	Quantitative	Northeast	1/1/2020	12/31/2021	1 054 465	20 283	No	No	Yes	No	No
Sadeghi et al,^116^ 2024	Quantitative	West	2/1/2021	9/30/2022	22 306	2238	No	Yes	No	No	No
Samuels-Kalow et al,^117^ 2024	Qualitative	Not specified	3/1/2021	5/31/2021	33	16	No	No	Yes	No	No
Shehan et al,^118^ 2021	Quantitative	Northeast	10/1/2019	4/10/2020	8023	1695	No	No	Yes	No	No
Smith et al,^119^ 2024	Quantitative	West	1/1/2019	12/31/2021	3172	566	No	No	Yes	No	No
Spierling Bagsic et al,^120^ 2023	Mixed methods	West	10/1/2017	3/30/2020	302	282	No	No	No	No	Yes
Steyer et al,^121^ 2023	Quantitative	Midwest	3/23/2020	9/24/2021	183	17	No	No	Yes	No	No
Sumarsono et al,^122^ 2023	Quantitative	South	3/1/2020	6/30/2022	2 639 284^g^	1 038 335^g^	No	No	Yes	No	No
Sun et al,^123^ 2022	Quantitative	South	11/1/2017	12/2/2020	5075	469	No	Yes	No	No	No
Szilagyi et al,^124^ 2020	Quantitative	West	8/1/2017	7/31/2018	39 871	849	No	Yes	No	No	No
Thomason et al,^125^ 2022	Quantitative	West	3/1/2019	3/31/2021	2945	334	No	No	Yes	No	No
Trombello et al,^126^ 2020	Quantitative	South	6/1/2015	7/31/2018	105	48	No	No	Yes	No	No
Turer et al,^127^ 2022	Quantitative	South	4/5/2021	4/4/2022	60 314^g^	2302^g^	No	Yes	No	No	No
Ugalde et al,^128^ 2023	Quantitative	Nationwide	3/1/2020	4/30/2021	569	140	No	No	Yes	No	No
Vázquez et al,^129^ 2024	Quantitative	Nationwide	8/24/2021	4/20/2022	511	236	No	No	Yes	No	No
Vahia et al,^130^ 2015	Quantitative	West	NA	NA	22	22	No	No	Yes	No	No
Vaughan et al,^27^ 2023	Quantitative	South	3/6/2019	12/31/2020	5410	914	No	No	Yes	No	No
Vaughan et al,^131^ 2024	Quantitative	South	1/13/2018	8/30/2022	134	134	No	No	No	No	Yes
Vehawn et al,^132^ 2014	Qualitative	West	NA	NA	27	27	No	No	No	Yes	No
Wallace et al,^133^ 2016	Quantitative	West	5/1/2012	4/30/2013	36 549	2272	No	Yes	No	No	No
Wang et al,^134^ 2022	Quantitative	Not specified	3/1/2019	2/28/2021	77 977	10 575	No	Yes	No	No	No
Wang et al,^135^ 2023	Quantitative	South	1/1/2019	12/31/2020	40 544	4603	No	Yes	No	No	No
Weiss-Laxer et al,^136^ 2022	Mixed methods	South	5/1/2020	9/30/2020	38	38	No	No	Yes	No	No
Xiong et al,^137^ 2021	Quantitative	Northeast	3/24/2019	5/18/2020	11 056	231	No	No	Yes	No	No
Yudkin et al,^138^ 2024	Quantitative	South	8/1/2020	4/30/2021	73	56	No	No	No	Yes	No

Abbreviations: mHealth, mobile health; NA, not available; RCTs, randomized clinical trials.

^a^ Included studies specifying *Spanish preferring* and *monolingual and bilingual*.

^b^ Included mobile applications (apps), text messaging, chat, digitally connected home health monitoring devices, social media, and telemonitoring.

^c^ When specified, included 21 instances of Epic; 2, Cerner; 1, Athena; and 1, custom (Kaiser).

^d^ Included synchronous video visits.

^e^ Included health-related websites, e-newsletters, and online learning modules.

^f^ Included more than 1 technology (eg, portal and telehealth).

^g^ Encounters, not unique individuals.

### Populations Studied

Specific diseases or associated risk were identified in 43 studies, of which 13 (30%) were most frequently type 2 diabetes,^39,45,47,48,60,62,78,85,103,104,113,120,131^ 10 (23%) were cancer,^39,41,50,63,73,95,96,108,111,114^ 4 (9%) were HIV,^37,74,82,105^ 4 (9%) were hypertension,^48,53,78,113^ 4 (9%) were asthma,^77,84,106,128^ and 3 (7%) were depression.^54,109,126^ Nine studies limited their eligible population by gender or sex^43,50,59,68,83,96,119,132,136^; of these, 7 (78%) focused on women.^50,59,68,96,119,132,136^ Of the 56 studies in which a health care setting was specified, 33 (59%) took place at an academic medical center,^36,37,38,44,45,46,55,57,59,61,63,67,70,71,80,95,98,99,101,106,108,112,115,116,117,118,119,121,123,124,126,127,136^ 15 (27%) at a federally qualified health care center or safety-net health system,^20,23,28,42,49,58,60,62,69,75,91,97,104,120,122^ and 8 (14%) at other locations (eg, a regional network or comprehensive cancer center).^26,65,66,82,85,92,93,111^

With respect to technology use, 15 of 106 studies (14%) limited their eligible population to technology users (eg, persons who completed at least 1 telehealth visit).^37,51,55,81,84,87,91,92,101,105,109,110,113,121,128^ All other studies evaluated participants eligible to use technology, new users, or patients of unknown technology-use backgrounds. Of 104 studies reporting a location or locations,^35^ 11 (11%) were in the Midwest,^26,36,41,53,69,71,75,81,104,113,121^ 23 (23%) were in the Northeast,^39,43,44,56,59,63,64,67,73,82,84,86,88,94,98,101,102,103,106,107,115,118,137^ 25 (25%) were in the South,^27,37,47,54,57,58,61,66,70,74,91,92,95,99,105,110,111,122,123,126,127,131,135,136,138^ 38 (37%) were in the West,^28,38,42,45,46,48,49,50,51,52,55,60,62,68,72,76,77,78,79,80,83,85,87,89,96,97,108,109,112,114,116,119,120,124,125,130,132,133^ and 7 (7%) were in multiple regions^40,65,90,93,100,128,129^ (Table 1).

### Barriers

We identified low overall and digital literacy,^39,43,74,77,84,90,94,105,106,117,132^ limited access to computers or mobile telephones,^43,70,88,96,97,106,110,117^ and inadequate broadband and mobile data plans as core barriers across technology categories.^26,28,43,59,86,88,97,101,110^ Other barriers, especially common among patient portal and telehealth studies, included lack of awareness,^26,61,77,84,106^ limited availability and appropriateness of patient-facing materials in Spanish,^61,73,77^ and lack of interpreter availability.^36,68,88,96,101^ Among the mHealth studies, reported barriers also included concerns associated with alert fatigue and information overload,^69^ data privacy,^53,61,103,105^ lack of a human connection,^96,105^ and lack of easy access to online resources.^132^ External factors not unique to Spanish speakers, including lower rates of being offered a portal account compared with English speakers^46^ and relative lack of Wi-Fi access,^115^ were also cited as digital health access barriers. Despite these inequities, there were no significant differences in Spanish compared with English speakers’ beliefs about portal effectiveness in enhancing care quality.^97^

### Facilitators

The most common facilitators across technology types were interventions that leveraged mobile devices,^38,40,41,42,45,53,54,60,62,78,83,94,120,131^ culturally and linguistically tailored content,^41,43,56,61,74,82,87,113^ and care partner engagement.^39,47,94,117^ For example, a study evaluating an app made for Spanish speakers identified information accessibility, attention to privacy concerns, and direct connections to care teams as key factors associated with better HIV medication adherence, mood and stress levels, and client–practitioner communication.^105^

Several studies involving portals, telehealth, and mHealth also demonstrated the value of digital health technologies in addressing, and in some cases overcoming, health care barriers, including inadequate culturally or linguistically tailored communications and educational materials,^42,50,52,83,102,103,106^ lower literacy,^105,117^ lower levels of acculturation,^50^ lack of trust,^48,104^ concerns associated with immigration status,^64^ lack of follow-up and care coordination,^64,69^ and barriers associated with excess emergency department use.^64^ One study found that despite being less likely to have a portal account, Spanish-speaking portal users messaged at similar rates as English speakers.^114^

The findings were mixed regarding Spanish speakers’ interest, satisfaction, and preference for telehealth compared with in-person visits, with studies demonstrating positive,^37,73,101,109,128^ negative,^51,66,71,79,118,121^ mixed,^89^ neutral,^76,137^ and uncertain^55,64^ results. Despite varied penetrance, there were 2 studies in which telehealth-related outcomes were better among Spanish speakers.^58,126^ One study reported that Spanish speakers preferred telehealth appointments from local community clinics where Spanish-speaking staff were available.^126^ Another study speculated that unawareness of access to Spanish-speaking personnel may have been associated with telehealth preference.^58^

### Technology Use Patterns

Most studies comparing digital health technology use with language focused on patient portals and telehealth. These studies revealed consistently lower portal account activation,^46,49,57,65,92,123,124^ portal use,^49,84,99,114,116,124,127,133,134,135^ telehealth adoption,^44,52,58,63,67,72,75,80,93,98,107,108,112,119,122,125,129^ and online resource use^100^ among Spanish speakers compared with English speakers. The methods and outcomes used to assess the magnitude of disparities varied widely, and a systematic evaluation of outcomes is beyond the scope of this review. In most studies of active portal users, Spanish-speaking patients were less likely to request medication refills,^133^ schedule or cancel appointments,^116^ communicate with care teams,^133^ view test results,^127^ and complete web-based or portal-based patient-reported outcome measures.^95,106,111^ However, 2 studies contradicted these trends. One study found that Spanish-speaking patients had higher odds of being new patient portal (MyChart, Epic Systems) adopters than English speakers (odds ratio, 1.56 [95% CI, 1.08-2.25]).^27^ Another study found that, despite being less likely to have a portal account, LEP portal users, most of whom were Spanish speakers, had similar rates of portal messaging for genetic counseling compared with English speakers; authors associated this with the accessibility of medical interpreters for counseling.^114^

### Intervention Outcomes

Several studies reported ubiquitous access to smartphones and frequent use of text messaging and social media among Spanish speakers.^50,61,110^ In a study on clinical trial recruitment, Spanish speakers strongly preferred text messaging over portal-based recruitment.^38^ In another study, social media recruitment attracted more participants who were Spanish speakers, had fewer years of US residency, were first-generation American, and were less likely to have health insurance.^40^ Three studies that were focused on text messaging–based diabetes interventions showed acceptability^45,62^ and efficacy^45,60^ compared with other modalities. mHealth interventions were successfully adopted among Spanish speakers with cancer,^41^ hypertension,^53,78^ diabetes,^78,131^ depression,^54^ HIV,^83^ and addiction.^94^ Other successful mHealth outcomes were associated with increased activity among patients who were sedentary,^42^ increased engagement in HIV prevention of young Hispanic or Latino immigrant men who were sexually active with men,^83^ remotely reported events in a diabetes self-management technology intervention,^85^ and improved hemoglobin A_1C_ levels among those with uncontrolled diabetes.^131^

On the other hand, telehealth compared with in-person studies did not reveal significantly different rates for Spanish speakers undergoing neurocognitive testing^130^ or well-child visits.^136^ Yudkin et al^138^ reported no significant outcome differences by language in their mHealth behavior change intervention, and Kafashzadeh et al^77^ reported that Spanish speakers with activated portal accounts did not experience a reduction in asthma severity.

## Discussion

In this scoping review, we identified persistent disparities in digital health access, use, and technology-based intervention outcomes between Spanish and English speakers in the US. Studies found that Spanish speakers were comparatively less likely to use patient portals, telehealth, or web-based resources; however, Spanish speakers widely used and were associated with beneficial outcomes from mHealth technologies, given nearly ubiquitous mobile device ownership and competency. When interventions were culturally tailored and integrated professional interpreters or care partners, Spanish-speaking patients were more likely to be successful at harnessing technologies for health management. Barriers among Spanish speakers were similar across technologies; several overlapped with systemic and socioeconomic barriers known to be associated with health outcomes. Other barriers that may have exacerbated digital health disparities among Spanish speakers included inadequate access to broadband and mobile data plans and low levels of overall literacy, health literacy, and digital literacy.

Studies evaluating patient portals showed lower rates of awareness, access, activation, and feature use among Spanish speakers; however, more nuanced feature-use patterns and needs, such as portal messaging patterns and content, remain incompletely defined and constitute a gap in the literature. Studies demonstrating Spanish speakers’ relative lack of awareness of patient portals and lower rates of portal invitations are consistent with findings from the Health Information National Trends Survey, which reported lower rates of clinicians offering or encouraging use of the portal to Black and Hispanic patients^139^; these trends suggest the need for marketing materials targeted to Spanish-speaking patients and educating clinicians and staff to increase invitation rates. Tailoring messaging to Spanish speakers was identified as particularly important for overcoming cultural barriers such as distrust in online health information and fear associated with deportation.^140,141^ This may be especially useful in clinical environments, such as emergency departments and federally qualified health centers, which often have fewer resources to put toward digital literacy training and marketing of digital health tools, that disproportionately treat immigrant Spanish speakers.^142^

Successful integration of certified interpreters was associated with the success or failure of telehealth interventions, whereas asynchronous communication technologies like the portal and text messaging were less dependent on the availability of interpreters in real time. While some device-related disparities have abated with the increasing ubiquity of mobile telephones, telehealth—particularly during the COVID-19 pandemic—highlighted disparities in Wi-Fi and data plan quality. Despite these challenges, patients and care partners identified telehealth as providing convenience,^96,104^ cost savings,^96,104^ infectious disease risk reduction,^96,104,117^ and potential to assist in reducing emergency department overuse and clinic no-shows.^64,69^

Studies focused on mHealth highlighted a strong preference among Spanish speakers to use text messaging as a primary intervention modality. Comparatively, English speakers responded positively to interventions delivered through the web, portal, telehealth, and text messaging. This finding may have account management implications for health systems and technology vendors, especially when some Spanish-speaking immigrant families may share a single device or mobile telephone number,^143^ and many families struggle to pay for sufficient data plans to support modern digital care. Spanish speakers’ preference for text messaging and apps and reduced access to data plans may have profound implications on their access to clinical trials and remote care services.

Privacy (especially surrounding immigration status) and linguistic and cultural tailoring were identified as key facilitators to a more user-centered design. Additionally, we identified care partner involvement as an important facilitator for adoption of multiple digital health technologies. Care partners have been identified as vital facilitators in other populations,^144^ particularly older adults^145^; this finding builds on that literature. Given that care partners may play a central role in the success of Spanish speakers’ digital health technology use, future interventions should incorporate care partners into marketing, education, and design.

### Future Directions

The issues raised in this scoping review should direct investigators, vendors, and health systems to the importance of local evaluation to understand how their workflows and designs address barriers and facilitate use among Spanish speakers. Further studies evaluating the penetrance and extent of Spanish language patient portal configurations and messaging workflows would provide valuable information for a national strategy. Vendors could work to further integrate text messaging workflows into their designs, given the strong preference for text messaging–based workflows among Spanish speakers. Finally, even if, as a society, we can overcome these existing barriers, new challenges are arising regarding artificial intelligence (AI), particularly generative AI models, which are rapidly integrating into electronic health record systems across the country. To our knowledge, no current literature exists evaluating patients’ awareness and comfort levels using portal-integrated AI and related tools.

### Limitations

This study has limitations. First, we may have excluded important studies given our inclusion criteria of US-based studies published in English. We also excluded conference abstracts and non–peer-reviewed literature, which may have provided additional insights into topics covered by our review. While our search terms yielded studies of multiple technologies, there may have been unreported digital health resources due to our search strategy. We also did not review references from all included papers, which may have yielded additional studies.

For the qualitative analysis, we focused on themes reported in the study objectives and key findings. Thus, there is a possibility that some findings may have been underreported. While coding open-ended text, we also noted a geographic bias. Studies were overrepresented from the Midwest (11%) and Northeast (23%) despite a limited proportion of the national Spanish-speaking population living in those regions (9.0% in the Midwest and 14.5% in the Northeast).^146^ Some communities with higher proportions of Spanish speakers may have unique facilitators that are not well represented in the literature. When reviewing inclusion criteria, identifying whether a study included adequate representation of Spanish-speaking participants required an element of reviewer judgment. In these instances, we enlisted a third reviewer to facilitate consensus.

Similarly, we were unable to apply a consistent definition of *Spanish speaker*, given that studies applied the term heterogeneously; instead, we relied on each study’s application. *Spanish speaking* and *Spanish preferring* were used interchangeably; most studies defined Spanish speaking by participant self-report or using the US Census Bureau’s definition of language spoken at home. We strove for consistency by reviewing the metadata to include individuals whose preferred language was Spanish (ie, not bilingual). The lack of a standardized nomenclature is a gap that may be addressed collaboratively to coalesce the field and facilitate comparative research. Lastly, while 72 of 106 studies (68%) were published within the past 5 years, few analyzed data collected after the 21st Century Cures Act’s information blocking rule of 2021.^147^

## Conclusions

The findings of this scoping review suggest that digital health technologies have become indispensable to health care delivery in the US. We provide a valuable summary of the current knowledge of digital health technologies among Spanish speakers. We identified disparities in use, primarily associated with limitations in literacy, awareness, consistent access to Wi-Fi and data plans, and interpreter availability. Facilitators included use of text messaging or social media–based interventions; integration of care partners and bilingual staff; and use of linguistically and culturally tailored marketing, content, and educational materials. In the future, digital technology interventions should integrate key facilitators, and research should evaluate the efficacy, safety, and acceptability of AI-assisted technologies such as machine learning to improve the availability, efficiency, and quality of translated content.

## Notes

Supplement 1. **eTable 1.** PRISMA ScR Checklist **eTable 2.** Literature Search Strategy **eFigure.** Covidence Extraction Template **eTable 3.** Thematic Analysis Codebook Exemplar (Population Characteristics) **eTable 4.** Thematic Analysis Codebook Exemplar (Key Findings)

Supplement 2.
Data Sharing Statement
