# Supplementary material for: Digital Health Technology Use Among Spanish Speakers in the US: A Scoping Review
Source: JAMA Netw Open. 2025 May 15;8(5):e2510386. doi: 10.1001/jamanetworkopen.2025.10386 (PMC12082372; doi:10.1001/jamanetworkopen.2025.10386)
Supplement: Supplement 1. — eTable 1. PRISMA ScR Checklist eTable 2. Literature Search Strategy eFigure. Covidence Extraction Template eTable 3. Thematic Analysis Codebook Exemplar (Population Characteristics) eTable 4. Thematic Analysis Codebook Exemplar (Key Findings) [file jamanetwopen-e2510386-s001.pdf]

Supplemental Online Content

Higashi RT, Thakur B, Repasky EC, et al. Digital health technology use among Spanish speakers in the US: a scoping review. *JAMA Netw Open*. 2025;8(5):e2510386. doi:10.1001/jamanetworkopen.2025.10386

eTable 1. PRISMA ScR Checklist
eTable 2. Literature Search Strategy
eFigure. Covidence Extraction Template
eTable 3. Thematic Analysis Codebook Exemplar (Population Characteristics)
eTable 4. Thematic Analysis Codebook Exemplar (Key Findings)

This supplemental material has been provided by the authors to give readers additional information about their work.

© 2025 Higashi RT et al. *JAMA Network Open*.

eTable 1. PRISMA ScR Checklist

Note: page numbers refer to clean (non-track change) version

SECTION	ITEM	PRISMA-ScR CHECKLIST ITEM	REPORTED ON PAGE #
**TITLE**			
Title	1	Identify the report as a scoping review.	1
**ABSTRACT**			
Structured summary	2	Provide a structured summary that includes (as applicable): background, objectives, eligibility criteria, sources of evidence, charting methods, results, and conclusions that relate to the review questions and objectives.	1
**INTRODUCTION**			
Rationale	3	Describe the rationale for the review in the context of what is already known. Explain why the review questions/objectives lend themselves to a scoping review approach.	2
Objectives	4	Provide an explicit statement of the questions and objectives being addressed with reference to their key elements (e.g., population or participants, concepts, and context) or other relevant key elements used to conceptualize the review questions and/or objectives.	2
**METHODS**			
Protocol and registration	5	Indicate whether a review protocol exists; state if and where it can be accessed (e.g., a Web address); and if available, provide registration information, including the registration number.	N/A
Eligibility criteria	6	Specify characteristics of the sources of evidence used as eligibility criteria (e.g., years considered, language, and publication status), and provide a rationale.	2-3, Figure
Information sources*	7	Describe all information sources in the search (e.g., databases with dates of coverage and contact with authors to identify additional sources), as well as the date the most recent search was executed.	2
Search	8	Present the full electronic search strategy for at least 1 database, including any limits used, such that it could be repeated.	2<br>eTable 2 in Supplement 1
Selection of sources of evidence†	9	State the process for selecting sources of evidence (i.e., screening and eligibility) included in the scoping review.	2-3, Figure
Data charting process‡	10	Describe the methods of charting data from the included sources of evidence (e.g., calibrated forms or forms that have been tested by the team before their use, and whether data charting was done independently or in duplicate) and any processes for obtaining and confirming data from investigators.	2-3,<br>eFigure in Supplement 1
Data items	11	List and define all variables for which data were sought and any assumptions and simplifications made.	3, eTables 3-4 in Supplement 1
Critical appraisal of individual sources of evidence§	12	If done, provide a rationale for conducting a critical appraisal of included sources of evidence; describe the methods used and how this information was used in any data synthesis (if appropriate).	Not done

© 2025 Higashi RT et al. *JAMA Network Open*.

SECTION	ITEM	PRISMA-ScR CHECKLIST ITEM	REPORTED ON PAGE #
Synthesis of results	13	Describe the methods of handling and summarizing the data that were charted.	3
**RESULTS**			
Selection of sources of evidence	14	Give numbers of sources of evidence screened, assessed for eligibility, and included in the review, with reasons for exclusions at each stage, ideally using a flow diagram.	4, Figure
Characteristics of sources of evidence	15	For each source of evidence, present characteristics for which data were charted and provide the citations.	Table 3
Critical appraisal within sources of evidence	16	If done, present data on critical appraisal of included sources of evidence (see item 12).	Not done
Results of individual sources of evidence	17	For each included source of evidence, present the relevant data that were charted that relate to the review questions and objectives.	4-10, including Tables 1-3
Synthesis of results	18	Summarize and/or present the charting results as they relate to the review questions and objectives.	7-10
**DISCUSSION**			
Summary of evidence	19	Summarize the main results (including an overview of concepts, themes, and types of evidence available), link to the review questions and objectives, and consider the relevance to key groups.	10
Limitations	20	Discuss the limitations of the scoping review process.	12
Conclusions	21	Provide a general interpretation of the results with respect to the review questions and objectives, as well as potential implications and/or next steps.	12
**FUNDING**			
Funding	22	Describe sources of funding for the included sources of evidence, as well as sources of funding for the scoping review. Describe the role of the funders of the scoping review.	13

© 2025 Higashi RT et al. *JAMA Network Open*.

eTable 2. Literature Search Strategy

Query Date	Database	Time Period	Search Term
7/21/23	PubMed	10 years	("Patient Portals"[Mesh] OR "Health Records, Personal"[Mesh] OR "Patient access to records"[Mesh] OR "telemedicine"[Mesh] OR "Consumer Health Informatics"[Mesh] OR "Access to Information"[Mesh] OR "patient portal") AND ("Healthcare disparities"[Mesh] OR "Limited English Proficiency"[Mesh] OR "Sociodemographic Factors"[Mesh] OR "Socioeconomic Factors"[Mesh] OR "Health Services Accessibility"[Mesh] OR "Health Status Disparities"[Mesh] OR "Multilingualism"[Mesh] OR "Vulnerable Populations"[Mesh] OR "Diversity, Equity, Inclusion"[Mesh] OR "Hispanic or Latino"[Mesh] OR "Communication Barriers"[Mesh] OR "Health literacy"[Mesh] OR "health equity"[Mesh] OR "Minority Groups"[Mesh] OR "Ethnicity"[Mesh] OR "Population Groups"[Mesh]) AND Spanish
7/21/23	Scopus	10 years	TITLE-ABS-KEY((TITLE-ABS-KEY ("Health Records" OR "Patient access to records" OR "telemedicine" OR "Consumer Health Informatics" OR "Access to Information" OR "patient portal")) AND (TITLE-ABS-KEY ("Healthcare disparities" OR "Limited English Proficiency" OR "Sociodemographic Factors" OR "Socioeconomic Factors" OR "Health Services Accessibility" OR "Health Status Disparities" OR "Multilingualism" OR "Vulnerable Populations" OR "Diversity, Equity, Inclusion" OR "Hispanic or Latino" OR "Communication Barriers" OR "Health literacy" OR "health equity" OR "Minority Groups" OR "Ethnicity" OR "Population Groups")) AND TITLE-ABS-KEY (Spanish))
7/21/23	Web of Science	10 years	TI=(("Health Records" OR "Patient access to records" OR "telemedicine" OR "Consumer Health Informatics" OR "Access to Information" OR "patient portal") AND ("Healthcare disparities" OR "Limited English Proficiency" OR "Sociodemographic Factors" OR "Socioeconomic Factors" OR "Health Services Accessibility" OR "Health Status Disparities" OR "Multilingualism" OR "Vulnerable Populations" OR "Diversity, Equity, Inclusion" OR "Hispanic or Latino" OR "Communication Barriers" OR "Health literacy" OR "health equity" OR "Minority Groups" OR "Ethnicity" OR "Population Groups") AND Spanish) OR AB=(("Health Records" OR "Patient access to records" OR "telemedicine" OR "Consumer Health Informatics" OR "Access to Information" OR "patient portal") AND ("Healthcare disparities" OR "Limited English Proficiency" OR "Sociodemographic Factors" OR "Socioeconomic Factors" OR "Health Services Accessibility" OR "Health Status Disparities" OR "Multilingualism" OR "Vulnerable Populations" OR "Diversity, Equity, Inclusion" OR "Hispanic or Latino" OR "Communication Barriers" OR "Health literacy" OR "health equity" OR "Minority Groups" OR "Ethnicity" OR "Population Groups") AND Spanish)) OR AK=(("Health Records" OR "Patient access to records" OR "telemedicine" OR "Consumer Health Informatics" OR "Access to Information" OR "patient portal") AND ("Healthcare disparities" OR "Limited English Proficiency" OR "Sociodemographic Factors" OR "Socioeconomic Factors" OR "Health Services Accessibility" OR "Health Status Disparities" OR "Multilingualism" OR "Vulnerable Populations" OR "Diversity, Equity, Inclusion" OR "Hispanic or Latino" OR "Communication Barriers" OR "Health literacy" OR "health equity" OR "Minority Groups" OR "Ethnicity" OR "Population Groups") AND Spanish))

© 2025 Higashi RT et al. *JAMA Network Open*.

8/3/23	Google Scholar	10 years	Search ("patient portal" and "Spanish"); review first 5 pages of results against final Covidence results; import any non-duplicative results
4/28/24	PubMed	7/22/23 – 4/28/24	("Patient Portals"[Mesh] OR "Health Records, Personal"[Mesh] OR "Patient access to records"[Mesh] OR "telemedicine"[Mesh] OR "Consumer Health Informatics"[Mesh] OR "Access to Information"[Mesh] OR "patient portal") AND ("Healthcare disparities"[Mesh] OR "Limited English Proficiency"[Mesh] OR "Sociodemographic Factors"[Mesh] OR "Socioeconomic Factors"[Mesh] OR "Health Services Accessibility"[Mesh] OR "Health Status Disparities"[Mesh] OR "Multilingualism"[Mesh] OR "Vulnerable Populations"[Mesh] OR "Diversity, Equity, Inclusion"[Mesh] OR "Hispanic or Latino"[Mesh] OR "Communication Barriers"[Mesh] OR "Health literacy"[Mesh] OR "health equity"[Mesh] OR "Minority Groups"[Mesh] OR "Ethnicity"[Mesh] OR "Population Groups"[Mesh]) AND Spanish

© 2025 Higashi RT et al. *JAMA Network Open*.

eFigure. Covidence Extraction Template

PREVIEW

Methods

Study Class

- ☐ Quantitative
- ☐ Qualitative
- ☐ Mixed Methods

Study Design

e.g. RCT, cohort study, interview, focus group, observation, usability, etc.

Study Population (i.e. core patient characteristics, clinical environment, patient type)

Study Setting

Core information such as:
- Urban/suburban/rural
- Region of country (a la Northeast, Midwest, South, West, Pacific)

https://www2.census.gov/geo/pdfs/maps-data/maps/reference/us_regdiv.pdf

Objective

Core questions, interventions, outcomes of the study. Will vary based on the design.

Study Start Date

Please use (MM/DD/YYYY).

i.e. 01/01/1987

Study End Date

Please use (MM/DD/YYYY).

i.e. 01/01/1987

Technology Used

(Choose all that apply)

- ☐ Patient portal
- ☐ Telehealth
- ☐ Text messaging
- ☐ Wearables
- ☐ Web-based resources
- ☐ Other

Results and Conclusions

Total Sample Size

Total Spanish Sample Size

Key Findings

Notes

<u>Definitions</u>:

- “Patient portal” included studies about the portal (e.g., Epic’s MyChart) or other personal health records
- “Telehealth” included synchronous audio-video or audio only communications
- “Text messaging”, “wearables” (e.g. Apple Watch, FitBit), and “other” mobile technologies such as apps, social media, and home health monitoring devices were later grouped under the category mobile health (“mHealth”)
- “Web-based resources” included educational or interventional content intended for use via a browser on a personal computer, rather than on a mobile device
- “Spanish sample” was documented using the article’s native definition, usually based on participant self-identification, and may have included individuals who were “Spanish speaking”, “Spanish preferring”, “monolingual/ speaks only Spanish”, or “speaks Spanish better than English”. We excluded: “English preferring” (if also spoke Spanish), “speaks English better than Spanish”, and “bilingual” persons of any age

© 2025 Higashi RT et al. *JAMA Network Open*.

eTable 3. Thematic Analysis Codebook Exemplar (Population Characteristics)

**Code**	**Definition**
Population	
age	target or eligible age of study population, if limited
gender	target or eligible gender of study population, if limited
other	other demographic characteristic defined for study population
Setting	
health system type	e.g., academic medical center, federally qualified healthcare center
clinic type	e.g., pediatrics, emergency department
other	e.g., community health fair
Eligibility criteria	
illness type	e.g., persons at risk for type 2 diabetes, persons with HIV
technology	e.g., persons who completed at least 1 telehealth appointment
other	e.g., persons who currently smoke
Notes	

© 2025 Higashi RT et al. *JAMA Network Open*.

eTable 4. Thematic Analysis Codebook Exemplar (Key Findings)

**Code**	**Definition**
barriers	barriers to technology use
facilitators, suggestions	facilitators, critiques, and ways to enhance technology
impacts, outcomes	outcomes of technology use or intervention, e.g., promoted healthy behavior; feasibility/acceptability intervention studies. e.g., "privacy a concern among Spanish speakers" = impact because it is an outcome
experiences, perceptions	positive or negative experiences, preferences, opinions, attitudes, perceptions. If about an outcome, code to impacts. e.g., "Spanish speakers were open to using telehealth for consult services"
descriptive	sociodemographic characteristics associated with a technology, e.g., telehealth users were more likely to be English-speaking

© 2025 Higashi RT et al. *JAMA Network Open*.
