# Supplementary material for: Digital Health Technology Use Among Spanish Speakers in the US: A Scoping Review
Source: JAMA Netw Open. 2025 May 15;8(5):e2510386. doi: 10.1001/jamanetworkopen.2025.10386 (PMC12082372; doi:10.1001/jamanetworkopen.2025.10386)
Supplement: Supplement 2. — Data Sharing Statement [file jamanetwopen-e2510386-s002.pdf]

Data Sharing Statement

Higashi. Digital Health Technology Use Among Spanish Speakers in the US. *JAMA Netw Open*. Published May 15, 2025. doi:10.1001/jamanetworkopen.2025.10386

Data
Data available: No

Additional Information
Explanation for why data not available: Given the nature of the study as a scoping review, all data utilized are available as previously published materials.
